# Bacteriocin from the Raccoon Dog Oral Microbiota Inhibits the Growth of Pathogenic Methicillin-Resistant Staphylococcus aureus

**DOI:** 10.32607/actanaturae.27349

**Published:** 2024

**Authors:** M. N. Baranova, E. A. Soboleva, M. A. Kornienko, M. V. Malakhova, Yu. A. Mokrushina, A. G. Gabibov, S. S. Terekhov, I. V. Smirnov

**Affiliations:** Shemyakin–Ovchinnikov Institute of Bioorganic Chemistry, Russian Academy of Sciences, Moscow, 117997 Russian Federation; Federal Research and Clinical Center of Physical-Chemical Medicine, Federal Medical Biological Agency, Moscow, 119435 Russian Federation; Faculty of Chemistry, Lomonosov Moscow State University, Moscow, 119991 Russian Federation

**Keywords:** ultra-high-throughput screening, antimicrobial resistance, antimicrobial peptides, lytic enzymes

## Abstract

The growing incidence of infections caused by antibiotic-resistant strains of pathogens is one of the key challenges of the 21^st^ century. The development of novel technological platforms based on single-cell analysis of antibacterial activity at the whole-microbiome level enables the transition to massive screening of antimicrobial agents with various mechanisms of action. The microbiome of wild animals remains largely underinvestigated. It can be considered a natural reservoir of biodiversity for antibiotic discovery. Here, the *Staphylococcus pseudintermedius *E18 strain was isolated from the oral microbiome of a raccoon dog (*Nyctereutes procyonoides*) using a microfluidic ultrahigh-throughput screening platform. *S. pseudintermedius* E18 efficiently inhibited the growth of pathogenic methicillin-resistant *Staphylococcus aureus *(MRSA). It was established that the main active substance of the *S. pseudintermedius *E18 strain was a bacteriocin with a molecular weight of 27 kDa. The identified bacteriocin had a high positive charge and an extremely narrow spectrum of activity. Bacteriocin *S. pseudintermedius *E18 was inactivated by elevated temperature, proteinase K, and EDTA. Further investigation on the structure of the bacteriocin produced by *S. pseudintermedius* E18 will provide a comprehensive understanding of its mechanism of action, which will open up prospects for developing novel DNA-encoded antimicrobials.

## DISCUSSION

Searching for novel antimicrobials is essential, since bacteria are constantly evolving and develop resistance to new antibiotics [1]. The application of novel platforms based on the metabolomics, genomic, and transcriptomic sequencing techniques, followed by bioinformatics analysis, as well as the drive toward alternative microbial culture methods offers new opportunities for antibiotic activity screening of naturally occurring substances.

Animal microbiomes are a unique reservoir that can be tapped in the search for novel antimicrobials [2, 3, 4]. Probiotic microorganisms are of special interest as potential producers of antibiotics [5, 6]. Although probiotic strains and commensal bacteria can have indirect implications on the microbiome by influencing the host immune system [7] or producing functionally important enzymes [8], the direct pathogen- killing mechanism is typical of most bacteriocins.

Here, we conducted ultra-high-throughput screening of the salivary microbiome of the raccoon dog (*Nyctereutes procyonoides*) to isolate strains producing substances exhibiting an antimicrobial activity against* S. aureus *and identify the metabolites responsible for their antagonistic properties.

**Fig. 1:**
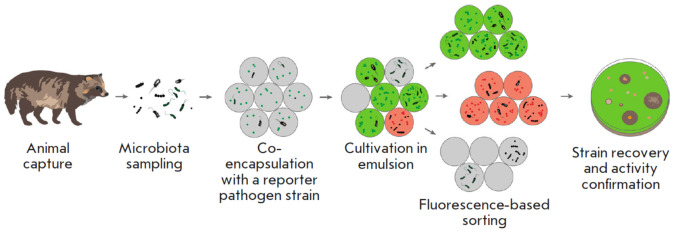
Schematic diagram of an ultra-high-throughput screening platform for selecting microorganisms that inhibit the growth of a target bacterium

Previously, we described a platform for ultrahigh- throughput screening of microbial communities (*Fig. 1*) [2, 9] that was based on cocultivation of individual microbial cells with a reporter pathogen strain in isolated droplets of double emulsion, followed by the isolation of active phenotypes by fluorescence-activated cell sorting. This platform was used to profile the oral microbiome of the raccoon dog and to identify the strains exhibiting an activity against *S. aureus*.

Screening revealed six phenotypically different strains reproducibly inhibiting the growth of *S. aureus* on a BHI agar medium and in liquid culture (activity was evaluated using the twofold serial dilution method). The strains were identified by matrixassisted laser desorption ionization time-of-flight mass spectrometry (MALDI-TOF MC) (*Table 1*).

**Fig. 2:**
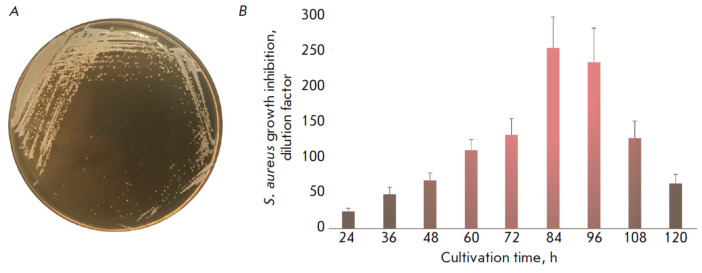
(*A*) The phenotype of the E18 producer strain on a BHI agar medium; (*B*) the dynamics of E18 strain active metabolite production

The *S. pseudintermedius *E18 strain exhibited the highest activity against *S. aureus *in the liquid medium (*Fig. 2A*). A more detailed analysis of the dynamics of the antagonistic effect of the producer strain was conducted to accumulate and identify the active substance (*Fig. 2B*).

**Table 1:** Mass spectrometric identification and antagonistic activity of the isolated strains against S. aureus

Strain	Microorganism	Growth inhibition zones of S. aureus, diameter, mm	Maximum activity in liquid culture	
			Inhibitory dilution factor	Cultivation duration, days
E14	Bacillus pumilus	11 ± 2	61 ± 9	2
E18	Staphylococcus pseudintermedius	5 ± 1	256 ± 47	4
E32	Bacillus amyloliquefaciens	4* ± 1	5 ± 1	2
EB10	Pasteurella dagmatis	0.9 ± 0.1	8 ± 1	8
EB16	Ralstonia insidiosa	3.1 ± 0.4	–	–
EB27	Curtobacterium luteum	–	4 ± 1	1
EB30	Brachybacterium sp.	–	2.1 ± 0.5	1

^*^Diffuse zone of inhibition.

The active substance was purified by solid-phase extraction using a LPS-500 sorbent (*Table 2*). Most of the substance could not be eluted by increasing the acetonitrile concentration in the buffer solution at pH 5.0; 0.1% trifluoroacetic acid (TFA) in an aqueous acetonitrile solution was used for elution.

**Table 2:** Purification of the substance produced by the E18 strain by pulsed solid-phase extraction using the LPS-500 sorbent

Buffer solution	A	B	A + C, %C		
			40	70	100
Activity according to inhibitory dilution, % of applied sample	2 ± 1	9 ± 1	12 ± 3	68 ± 7	11 ± 2

Note. Buffer A: 10 mM NH4OAc, 5% acetonitrile, pH 5.0; buffer B: 10 mM NH4OAc, 80% acetonitrile, pH 5.0; buffer C: 0.1% TFA, 80% acetonitrile.

The ... 4

As a result of 1.5 h of incubation at 60°C, the substance produced by the E18 strain lost its antibiotic activity. Because of thermal lability and the elution pattern during solid-phase extraction, we assumed it to be a high-molecular-weight substance. Active samples of the culture medium supplemented with 50 mM sodium phosphate, pH 7.5, were exposed to proteinase K (0.1 mg/mL). After 3 h of incubation at 37°C, the inhibitory activity against *S. aureus *was completely lost. Since the compound tentatively had a protein nature, the respective methods were used for its further purification.

The first purification stage involved ion exchange chromatography using the SP Sepharose sorbent (*Table 3*).

**Table 3:** Purification of the substance produced by the E18 strain by cation exchange chromatography using the SP Sepharose sorbent

Content of buffer B, %	0	20	40	60	80	100
Activity according to inhibitory dilution, % of applied sample	6 ± 5	2 ± 1	15 ± 3	26 ± 7	21 ± 5	11 ± 2

Note. Buffer A: 10 mM NH4OAc, pH 6.0; buffer B: 10 mM NH4OAc, 1 M NaCl, pH 6.0. Fractions corresponding to the 60 and 80% of buffer B content (600 and 800 mM NaCl, respectively) were used further in the work.

**Fig. 3:**
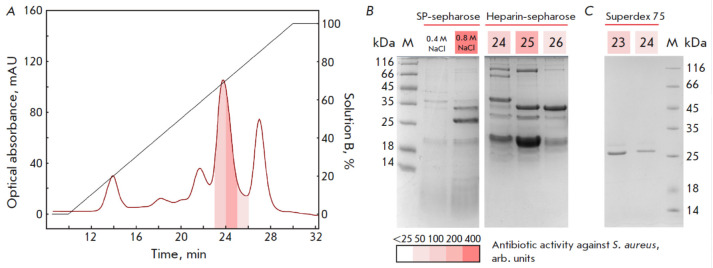
Chromatographic purification of the protein responsible for the activity of the *S. pseudintermedius *E18 strain. Fractions with the highest antistaphylococcal activity are shown in red. (*A*) Representative chromatogram obtained by fractionation of active metabolites of *S. pseudintermedius *E18 using a Heparin Sepharose column. (*B*) Representative 15% SDS-PAGE patterns. Protein purification by chromatography using the SP Sepharose sorbent and subsequent purification using a Heparin Sepharose column. (*C*) Representative 15% SDS-PAGE pattern. Fractions obtained as a result of protein purification by gel filtration using a Superdex 75 column

A Heparin Sepharose chromatography column (GE Healthcare, USA) and buffer solutions A (20 mM HEPES, pH 7.0) and B (20 mM HEPES, 1 M NaCl, pH 7.0), with a flow rate of 1 mL/min, were used at the second purification stage. Linear gradient elution with buffer B was performed for 20 min (*Fig. 3 A,B*). The retention time indirectly indicated that the protein carried a high positive charge.

Size exclusion chromatography using a Superdex 75 column in buffer solution containing 20 mM HEPES and 250 mM NaCl (pH 7.0, flow rate, 0.4 mL/min) was employed at the third purification stage. Activity corresponded to the protein ~ 27 kDa in size (*Fig. 3C*); retention time was ~ 23 min.

A purified protein was used for the functional studies. The minimum inhibitory concentration of this protein against *S. aureus *was 0.05 ± 0.02 µg/mL. The resulting bacteriocin was highly specific: its MIC values against *Escherichia coli*, *Pseudomonas aeruginosa*,* Klebsiella pneumoniae*, *Enterococcus faecium*,* Acinetobacter baumannii*, *Enterobacter cloacae*,* Streptococcus pneumoniae*, and *Bacillus cereus *were above 10 µg/mL, indication that it exhibited no antimicrobial activity against these bacteria.

Hence, the *S. pseudintermedius* strain produces class III bacteriocin, the thermolabile 27 kDa polypeptide inhibiting the growth of bacteria belonging to the Table 2. Purification of the substance produced by the E18 strain by pulsed solid-phase extraction using the LPS-500 sorbent Buffer solution A B A + C, %C 40 70 100 Activity according to inhibitory dilution, % of applied sample 2 ± 1 9 ± 1 12 ± 3 68 ± 7 11 ± 2 Note. Buffer A: 10 mM NH4OAc, 5% acetonitrile, pH 5.0; buffer B: 10 mM NH4OAc, 80% acetonitrile, pH 5.0; buffer C: 0.1% TFA, 80% acetonitrile. Fig. 2. (*A*) The phenotype of the E18 producer strain on a BHI agar medium; (*B*) the dynamics of E18 strain active metabolite production* A B S. aureus *growth inhibition, dilution factor Cultivation time, h 24 36 48 60 72 84 96 108 120 300 250 200 150 100 50 0 genus *Staphylococcus *[10, 11, 12]. Class III bacteriocins include bacteriolysins, tailocins, and nonlytic proteins. Bacteriolysins are the best studied subclass of such organisms. The known members of bacteriolysins are metal-dependent proteases catalyzing the hydrolysis of peptide bridges or stem peptides in peptidoglycan in a target bacterium [12]. A hypothesis was put forward that the protein isolated from *S. pseudintermedius* is also a peptidoglycan hydrolase. Incubation of the protein in the presence of 10 mM EDTA at 25°C for 15 min rendered it completely inactive. Therefore, the substance mediating the antistaphylococcal activity of the *S. pseudintermedius *strain is a metal-dependent enzyme, which is typical of bacteriolysins.

## CONCLUSIONS

The development of antimicrobial resistance by pathogenic bacteria has revived interest in antimicrobial agents that can target bacterial membranes and cell walls [13]. Ultra-high-throughput screening of the microbiota of a racoon dog uncovered the *S. pseudintermedius* E18 strain. It was demonstrated by chromatographic fractionation that this strain produces an antimicrobial agent acting as a lytic enzyme.
